# Role of the p38 MAP kinase pathway in *C. elegans* surface antigen switching

**DOI:** 10.17912/micropub.biology.000130

**Published:** 2019-07-04

**Authors:** Samuel M. Politz

**Affiliations:** 1 Department of Biology and Biotechnology, Worcester Polytechnic Institute, Worcester, MA

**Figure 1.**
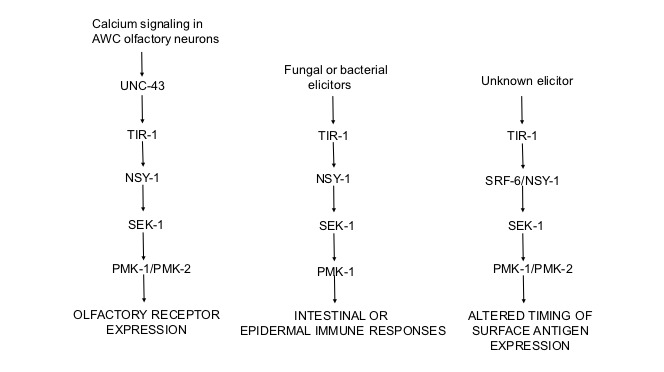
Involvement of the p38 MAP kinase pathway in three different processes in *C. elegans.* The p38 pathway *per se* consists of a MAPKKK (NSY-1), a MAPKK (SEK-1), and a MAPK (PMK-1). On the left, the p38 pathway is being used to determine cell fate in the AWC chemosensory neuron pair (Troemel, Sagasti, and Bargmann 1999). in the center, the p38 pathway is being used to regulate immune responses to fungal and bacterial pathogens. These include immune responses that occur in the epidermis or the intestine (reviewed in Partridge, Gravato-Nobre, and Hodgkin 2010). On the right, the p38 pathway is being used to regulate expression of an L1-specific surface epitope recognized by a monoclonal antibody (Foley et al., 2019). In addition to the MAP kinase cascade, *C. elegans* processes mediated by the p38 pathway utilize upstream signaling proteins such as the adapter protein TIR-1, as well as G proteins, phospholipase C, and protein kinase C (not shown, reviewed in Partridge, Gravato-Nobre and Hodgkin 2010).

## Description

In Van Sciver et al, 2019 and Honzel et al, 2019, we showed that the gene previously described as *srf-6* is actually *nsy-1*, which encodes NSY-1, the MAPKKK in the *C. elegans* p38 pathway. In earlier work, we had shown that *srf-6* mutations affect timing of expression of a surface epitope (Hemmer et al., 1991). Wild-type worms express the epitope only at the L1 stage, but *srf-6* mutants express it additionally at stages L2-L4 (called CLD for Constitutive Larval Display). In addition, we reported that wild-type worms can display the L1-specific epitope on stages L2-L4 when grown on a modified medium containing the concentrated extract of liquid nematode culture medium (called ILD for Inducible Larval Display, Grenache et al., 1996). Thus the expression of the L1-specific epitope appears to be controlled by an inducible switch that is under control of the *srf-6* gene, which, as we now know, is a component of the p38 MAP kinase pathway in *C. elegans.* The *srf-6* mutant phenotype, according to this model, corresponds to a switch that is constitutively “on”. *srf-6(yj13)* has a CLD phenotype similar to that of a large *nsy-1* deletion, suggesting that SRF-6 may function to inhibit expression of the L1-specific epitope after the L1 stage.

The modulation of this switch by an extract of liquid nematode culture medium (Grenache et al., 1996) suggested to us that it might be triggered by environmental signals detected by the nematodes’ chemical senses. Genes such as *daf-4* and *daf-7* encode components of a TGF beta pathway that control formation of the *C. elegans* dauer larva in response to dauer pheromone, which is secreted by worms and detected by worm chemosensation (reviewed in Patterson and Padgett 2000). *Daf-4* and*daf-7* mutants also show the CLD phenotype (Grenache et al 1996). This led us to test *srf-6* mutants for chemosensory defects directly (Olsen et al 2007). We determined that *srf-6(yj13)* mutants are defective in chemotaxis to both water-soluble and volatile attractants. Conversely, we also tested chemosensory mutants for ILD and found that genes required for integrity of the chemosensory ciliated nerve endings are also required for ILD (Olsen et al 2007). However, genes required for olfaction were not required for ILD. We note that *nsy-1* is expressed in other neurons in addition to AWC (Sagasti et al 2001), and is required in the ADF amphid neurons for pathogen-induced induction of serotonin biosynthesis (Shivers et al 2009). The tissue of expression and time of action of *nsy-1/srf-6* in relation to ILD remain to be determined.

A clue as to how *srf-6* might modulate surface antigen expression is found in the fact that neither *srf-3(yj10)* nor *srf-3(yj10)srf-6(yj43)* double mutants show immunofluorescence of an L1-specific epitope at any developmental stage (Hemmer et al 1991). The *srf-3* gene encodes a nucleotide sugar transporter, and the pathogenic bacteria *Yersinia* and *Microbacterium nematophilum* are unable to infect *srf-3* mutants (Hoflich et al 2004). Furthermore, *srf-3* mutants are deficient in glycoconjugates (Cipollo et al 2004). Thus *srf-6*might control the expression of specific glycosylation enzymes via sensing of environmental chemical conditions.

It is well established that the outer surface of parasitic nematodes is covered in a glycoprotein surface coat. Similarities can be found between the L1-specific epitope of *C. elegans* and the stage-specific expression of parasitic nematode “excretory-secretory antigens”. These are also found in association with the surface of the parasite. A good example is the *Toxocara canis*infective larva (L3), which has a glycoprotein coat composed primarily of an abundant mucin-like protein, TES120 (Page and Maizels 1992; Page, Rudin, and Maizels 1992). TES120 can also be found in the culture medium as a secretory product of this developmental stage (Page and Maizels 1992). The parasite has the ability to shed its TES120-containing surface coat in response to antibody binding (Smith et al 1981). We have found that mAb M37 staining is only visible on the surface of the *C. elegans* L1 when worms are incubated with the antibody at 0-4° C. When the sample is allowed to warm on the microscope stage, worms shed large fluorescent flakes or patches and eventually appear completely unstained (Politz and Philipp 1992). Thus a *C. elegans* surface epitope shows similar stage-specificity and ability to be released, as do surface coat molecules of parasitic nematodes. This raises the possibility that stage-specificity of the surface antigens of parasitic nematodes might also be controlled by a MAP kinase pathway.

## Reagents

*C. elegans* strains used in this work were listed in Van Sciver et al., 2019, Honzel et al., 2019, and Foley et al., 2019. Strains will be sent to the CGC.

## References

[R1] Cipollo JF, Awad AM, Costello CE, Hirschberg CB (2004). srf-3, a mutant of Caenorhabditis elegans, resistant to bacterial infection and to biofilm binding, is deficient in glycoconjugates.. J Biol Chem.

[R2] Foley Stephen J., Wu Zheyang, Politz Samuel M. (2019). A *C. elegans* MAP kinase pathway is required for wild-type display of an L1-specific surface antigen (*srf-6* is *nsy-1* III). microPublication Biology.

[R3] Grenache DG, Caldicott I, Albert PS, Riddle DL, Politz SM (1996). Environmental induction and genetic control of surface antigen switching in the nematode Caenorhabditis elegans.. Proc Natl Acad Sci U S A.

[R4] Hemmer RM, Donkin SG, Chin KJ, Grenache DG, Bhatt H, Politz SM (1991). Altered expression of an L1-specific, O-linked cuticle surface glycoprotein in mutants of the nematode Caenorhabditis elegans.. J Cell Biol.

[R5] Höflich J, Berninsone P, Göbel C, Gravato-Nobre MJ, Libby BJ, Darby C, Politz SM, Hodgkin J, Hirschberg CB, Baumeister R (2004). Loss of srf-3-encoded nucleotide sugar transporter activity in Caenorhabditis elegans alters surface antigenicity and prevents bacterial adherence.. J Biol Chem.

[R6] Honzel Brooke E., Foley Stephen J., Politz Samuel M. (2019). *C. elegans srf-6* and *nsy-1* mutations result in a similar 2AWC^ON^ phenotype and do not complement (*srf-6* is *nsy-1* II). microPublication Biology.

[R7] Olsen DP, Phu D, Libby LJ, Cormier JA, Montez KM, Ryder EF, Politz SM (2006). Chemosensory control of surface antigen switching in the nematode Caenorhabditis elegans.. Genes Brain Behav.

[R8] Page AP, Maizels RM (1992). Biosynthesis and glycosylation of serine/threonine-rich secreted proteins from Toxocara canis larvae.. Parasitology.

[R9] Page AP, Rudin W, Maizels RM (1992). Lectin binding to secretory structures, the cuticle and the surface coat of Toxocara canis infective larvae.. Parasitology.

[R10] Partridge FA, Gravato-Nobre MJ, Hodgkin J (2010). Signal transduction pathways that function in both development and innate immunity.. Dev Dyn.

[R11] Patterson GI, Padgett RW (2000). TGF beta-related pathways. Roles in Caenorhabditis elegans development.. Trends Genet.

[R12] Politz SM, Philipp M (1992). Caenorhabditis elegans as a model for parasitic nematodes: a focus on the cuticle.. Parasitol Today.

[R13] Sagasti A, Hisamoto N, Hyodo J, Tanaka-Hino M, Matsumoto K, Bargmann CI (2001). The CaMKII UNC-43 activates the MAPKKK NSY-1 to execute a lateral signaling decision required for asymmetric olfactory neuron fates.. Cell.

[R14] Shivers RP1, Kooistra T, Chu SW, Pagano DJ, and Kim DH. (2009) Tissue-specific activities of an immune signaling module regulate physiological responses to pathogenic and nutritional bacteria in C. elegans. Cell Host Microbe. 6: 321-30. 10.1016/j.chom.2009.09.001 PMC2772662 19837372

[R15] Smith HV, Quinn R, Kusel JR, Girdwood RW (1981). The effect of temperature and antimetabolites on antibody binding to the outer surface of second stage Toxocara canis larvae.. Mol Biochem Parasitol.

[R16] Troemel ER, Sagasti A, Bargmann CI (1999). Lateral signaling mediated by axon contact and calcium entry regulates asymmetric odorant receptor expression in C. elegans.. Cell.

[R17] Van Sciver Nicholas D., Pulkowski Jennifer O., Politz Samuel M. (2019). Three *C. elegans*
*srf-6* mutants carry *nsy-1* mutations (*srf-6* is *nsy-1* I). microPublication Biology.

